# Suicide risk, hopelessness, interpersonal needs, and mental health in a sample of migrant psychiatric patients: a case-control study

**DOI:** 10.1192/j.eurpsy.2024.1275

**Published:** 2024-08-27

**Authors:** L. Polidori, I. Berardelli, S. Sarubbi, G. Sarli, C. Corti, D. Erbuto, M. Pompili, M. Cifrodelli

**Affiliations:** ^1^Psychiatry Residency Training Program, Faculty of Medicine and Psychology; ^2^ Suicide Prevention Center, Sant’Andrea Hospital; ^3^Faculty of Medicine and Psychology, Sapienza University of Rome, Rome, Italy; ^4^International Consortium for Mood & Psychotic Disorder Research, McLean Hospital, Belmont, United States

## Abstract

**Introduction:**

Suicide is a multifactorial phenomenon characterized by many biological, psychological, and social-cultural factors. The study of this phenomenon in migrants is complex, with no theoretical framework that can describe the available heterogeneous data. Although Italy has the fourth largest migrant population of EU, only few studies have assessed suicidal risk in migrants.

**Objectives:**

The aim of his study is to assess suicide risk factors (hopelessness; interpersonal needs; traumatic experiences) in a sample of migrant patients, and to evaluate the possible application of the Interpersonal Theory of Suicide (ITS). Moreover, suicidal ideation and attempts were compared between migrants and natives. Lastly, a wider psychometric assessment has been conducted (depressive and anxiety symptoms; autistic traits).

**Methods:**

In this case-control study, we included 50 migrants vs. 50 natives. Data were collected during the same period by gender, age, and diagnosis. We collected sociodemographic and clinical characteristics. We administered the following tests: Columbia Suicide Severity Rating Scale, Interpersonal Needs Questionnaire, Beck Hopelessness Scale, Beck Depression Inventory-II, Hamilton Anxiety Scale, Childhood Trauma Questionnaire, and Adult Autism Subthreshold Spectrum.

**Results:**

There were no differences in sociodemographic characteristics, except for ethnicity. Otherwise, there were significative differences between diagnosis (p:0.013), with native reporting more Mood Disorders, and migrants reporting more Anxiety, Obsessive-Compulsive, Trauma-Related, Eating, and Substance Use Disorders. Migrants were more prone to be on treatment with Mood Stabilizers (p:0.000). There were significative differences for interpersonal needs, trauma, anxiety, and autistic traits. Migrants show more perceived burdensomeness (p:0.05), more physical neglect (p:0.004), physical abuse (p:0.002), and sexual abuse (p:0.016), more anxiety symptoms (p:0.046), and more empathy alterations (p:0.014). No differences were found for suicidal ideation and attempts, hopelessness, and depressive symptoms.

**Conclusions:**

Despite there were no differences in suicide risk, migrants showed higher rates of perceived burdensomeness (PB) and childhood traumatic experiences (CTE). Both PB and CTE represent cardinal constructs of the ITS. No differences were found for hopelessness and depressive symptoms. Migrants showed higher rates of anxiety symptoms and empathy alterations. Even if suicide rates between migrants and natives were similar, accurate assessment of suicidal risk in migrants is crucial in improving suicide prevention strategies. Suicide risk evaluation in migrants should consider the application of ITS. For an appropriate clinical evaluation of the migrant patients, anxiety dimensions and autistic traits should be investigated.

**Disclosure of Interest:**

None Declared

